# SLCO4A1-AS1 Facilitates the Malignant Phenotype via miR-149-5p/STAT3 Axis in Gastric Cancer Cells

**DOI:** 10.1155/2021/1698771

**Published:** 2021-10-19

**Authors:** Qing Li, Dachuan Zhang, Hui Wang, Jun Xie, Lei Peng, Yan Peng, Xiao Zheng, Jingting Jiang

**Affiliations:** ^1^Department of Pathology, The Third Affiliated Hospital of Soochow University, Changzhou, China; ^2^Department of Tumor Biological Treatment, The Third Affiliated Hospital of Soochow University, Jiangsu Engineering Research Center for Tumor Immunotherapy, Institute of Cell Therapy, Soochow University, Changzhou, China

## Abstract

Solute carrier organic anion transporter family member 4A1 (SLCO4A1-AS1), a newly discovered lncRNA, may exert effects in tumors. Since its role in gastric cancer remains obscure, we sought to explore the mechanism of SLCO4A1-AS1 in gastric cancer. The relationship among SLCO4A1-AS1, miR-149-5p, and STAT3 was detected by bioinformatics, dual luciferase analysis, and *Pearson*'s test, and the expressions of these genes were determined by quantitative real-time PCR and Western blot. Moreover, CCK-8, flow cytometry, wound healing assay, and Transwell analysis were performed to verify the function of SLCO4A1-AS1 in gastric cancer. Rescue experiments were used to detect the role of miR-149-5p. The expressions of SLCO4A1-AS1 and STAT3 were increased, while the expression of miR-149-5p was suppressed in gastric cancer tissues and cell lines. In addition, STAT3 expression was negatively correlated with miR-149-5p expression but was positively correlated with SLCO4A1-AS1 expression. Overexpression of SLCO4A1-AS1 promoted cell viability, migration, invasion, and STAT3 expression but suppressed apoptosis, while knockdown of SLCO4A1-AS1 had the opposite effect. SLCO4A1-AS1 bound to miR-149-5p and targeted STAT3. Moreover, miR-149-5p mimic inhibited the malignant development of gastric cancer cells and obviously reversed the function of SLCO4A1-AS1 overexpression. Our research reveals that abnormally increased SLCO4A1-AS1 expression may be an important molecular mechanism in the development of gastric cancer.

## 1. Introduction

Gastric cancer is a common cancer of the digestive tract [1]. Although the morbidity of gastric cancer has declined in recent years, its mortality rate is still among the highest in the world [2]. Diagnosis of the disease in the early stage together with certain treatment would help cure or prolong the survival of the patients; however, conventional treatment would be difficult or fail to treat patients when they are first diagnosed at advanced gastric cancer stage [3]. Therefore, the study of new mechanisms would provide new ideas for the treatment of gastric cancer.

Noncoding RNA (ncRNA) has recently become a research hotspot in the molecular mechanisms of new diseases [4]. Studies have shown that the occurrence and development of gastric cancer are accompanied by a variety of ncRNA expression disorders, and abnormally expressed ncRNAs will eventually affect epigenetics and lead to the formation of tumor malignant phenotypes [5, 6]. For example, the first studied microRNAs (miRNAs), such as miR-17-5p and miR-623, have shown significant regulatory effects on the proliferation, migration, and invasion of gastric cancer cells [7]. Circular RNA (circRNA) is a kind of ncRNA characterized by a closed covalent loop structure. Studies have found that circRNA has a more stable structure and can be detected through blood pathways (such as hsa_circ_0000745 and hsa_circ_0000419), which can guide the diagnosis and prognosis of gastric cancer to a certain extent [8, 9]. Of course, long noncoding RNA (lncRNA) is also an important part of the progression of gastric cancer. Zhang et al. screened and analyzed lncRNA of tissue samples from gastric cancer patients and adjacent normal tissues and then found that the expressions of plasma lncRNAs (TINCR, CCAT2, AOC4P, BANCR, and LINC00857) were significantly upregulated in gastric cancer cell lines [10]. Wang et al. found that lncRNA UCA1 regulates the stability of GRK2 protein by promoting Cbl-c-mediated G protein-coupled receptor kinase 2 ubiquitination, thereby increasing the metastatic ability of gastric cancer cells [11]. This also reflects that lncRNAs may become potential markers and possible targets for gastric cancer diagnosis and tumor treatment.

Solute carrier organic anion transporter family member 4A1 (SLCO4A1) is a newly discovered lncRNA in recent years. It is located on the long arm of chromosome 20 and contains 1440 bp in full length [12]. Most of the research on SLCO4A1 focuses on colon cancer. Some studies have found that the sense chain of SLCO4A1-AS1, SLCO4A1, belongs to the superfamily of membrane transport systems, which may serve as a valuable marker for poor prognosis of colorectal cancer. It also plays an important role in colorectal cell proliferation, migration, invasion, and carcinogenesis [13, 14]. In addition, SLCO4A1-AS1 also showed obvious cancer-promoting potential in bladder cancer and lung adenocarcinoma [15, 16]. However, there is no research evidence about SLCO4A1-AS1 in gastric cancer.

More and more studies have shown that lncRNAs can regulate multiple pathways through miRNAs to regulate the progress of gastric cancer [17–19]. For example, CASC11 acts as a ceRNA by sponging miR-340-5p to regulate cell cycle signals and affects the growth and apoptosis of gastric cancer cells [20]. However, it is not clear whether SLCO4A1-AS1 can also be used as ceRNA, and binding miRNA plays a regulatory role in gastric cancer. Therefore, we tried to explore the role of SLCO4A1-AS1 in gastric cancer and the possible regulatory pathways.

## 2. Materials and Methods

### 2.1. Ethics Statement and Tissue

The collection of clinical tissues was approved by the Ethics Committee of the Third Affiliated Hospital of Soochow University (ZC201903024), and the informed consent signed by the patients was obtained. The 46 pairs of tissue samples were all from pathologically and histologically confirmed gastric cancer patients who received treatment in our hospital from Apr 2019 to May 2020.

### 2.2. Cell and Culture

GES-1 human normal gastric mucosal epithelial cells (HTX1964) produced by Shenzhen Luxury Development Biotechnology Co., Ltd. (China) and other gastric cancer cell clines were cultured in Dulbecco's Modified Eagle's Medium (DMEM) (11960069, Gibco, USA) containing 10% Fetal Bovine Serum (FBS, 16140071, Gibco) in an incubator at 37°C with 5% CO_2_ and 100% humidity (MCO-20AIC, SANYO, Japan). AGS (ATCC® CRL-1739), SNU-16 (ATCC® CRL-5974), NCI-N87 (ATCC® CRL-5822), SNU-5 (ATCC® CRL-5973), and RF-48 (ATCC® CRL-1863) were produced by American Type Culture Collection (ATCC, USA).

### 2.3. Transfection

The recombinant plasmids for overexpressed SLCO4A1-AS1 (pc-SLCO4A1-AS1) and siRNA targeted SLCO4A1-AS1 (si-SLCO4A1-AS1) were purchased from Guangzhou GENESEED Biotechnology Co., Ltd. miR-149-5p mimic (B01001), miR-149-5p inhibitor (B03001), mimic-control (B04001), and inhibitor-control (B04003) were purchased from Shanghai GenePharma Company (China). AGS or SNU-16 cells (1 × 10^4^ cell/ml) cultured in 96-well plates were transfected with recombinant plasmids in accordance with Lipofectamine 3000 Transfection Kit (L3000001, Invitrogen, USA). After 24 h of cell transfection, total RNA was extracted to detect the transfection efficiency of plasmids using real-time quantitative PCR (RT-qPCR).

### 2.4. RT-qPCR

The total RNA from clinical tissues or gastric cancer cells was isolated using TRIzol reagent (15596018, Invitrogen, USA). The synthetization of complementary DNA was performed in the PrimeScript RT Reagent Kit (RR047A) from TaKaRa (Japan). RT-qPCR test was constructed in Mx3000P real-time PCR system (Agilent, USA) using Quant One-Step qRT-PCR Kit (FP303, TIANGEN, China) according to the following condition: 95°C predenaturation for 10 min, followed by a total of 40 cycles, denaturation (95°C, 15 sec), and annealing (60°C, 1 min). The mRNA expressions of target genes were analyzed by the 2^−ΔΔCt^ method [21], with GAPDH or U6 as control. The primers were listed as follows (5′-3′): SLCO4A1-AS1: (CTGGGCTGACAAGTGAGGAG, GGGTTCAAGTCAGCGACACT); STAT3: (CAGCAGCTTGACACACGGTA, AAACACCAAAGTGGCATGTGA); GAPDH: (GGGAAACTGTGGCGTGAT, GAGTGGGTGTCGCTGTTGA); miR-149-5p: (TCACTCCCGTCGTATCCAGT, GTATCCAGTGCGTGTCGTGG); U6: (AAAGCAAATCATCGGACGACC, GTACAACACATTGTTTCCTCGGA).

### 2.5. Cell Counting Kit-8 (CCK-8)

CCK-8 Kit (C0037) was purchased from Beyotime (China) to detect the cell viability in AGS or SNU-16 cells. After being cultured routinely for 24, 48, or 72 h, 10 μl of CCK-8 reagent was added to culture well. Then the AGS or SNU-16 cells were continuously cultivated for 2 h. Lastly, Microplate Absorbance Reader (E0228) from Beyotime (China) was employed to detect the absorbance (450 nm).

### 2.6. Flow Cytometry

The changes of cell apoptosis were detected by flow cytometry according to Annexin V-FITC Apoptosis Detection Kit (CA1020, Solarbio, China). After digestion with trypsin, AGS or SNU-16 cells were collected, followed by suspending the cells with 1 ml of 1 ×  binding buffer to bring the cell density to 1 × 10^6^ cells/ml. 100 μl of cells (1 × 10^5^ cells) was added to each tube, and 5 μl of Annexin V-FITC was added to the tube (room temperature, protected from light, and gently mixed for 10 min). Lastly, the cell mixture was performed by flow cytometer (CytoFLEX, Backman Coulter, USA) within 1 h to detect cell apoptosis.

### 2.7. Wound Healing Assay

AGS or SNU-16 cells (3 × 10^5^ cells/ml) were seeded into 6-well plates and cultured to 100% cell fusion. 100 μl pipette tip was used to draw a line wound through the layer of fused cells. After washing with PBS, the culture plate was cultured in serum-free medium for 24 h. At last, the wound closure was photographed and measured by the BX53M microscope from Olympus (Japan) (magnification ×100).

### 2.8. Transwell

Transwell chamber (3422, Corning, USA) precoated with Matrigel (354230, BD, USA) was inserted into a 24-well culture plate. The AGS or SNU-16 cell suspension and the medium without FBS were mixed and added to the upper chamber and then cultured for 24 h. Subsequently, after being fixed with methanol, the cells on the membrane were stained with 0.1% crystal violet solution (C8470, Solarbio, China). The cells on the membrane were photographed under a BX53M microscope (magnification ×200).

### 2.9. Target Relationship Prediction and Verification

The binding relationship of SLCO4A1-AS1 and miR-149-5p, miR-149-5p, and STAT3 was predicted by LncBase Predicted v.2 (http://carolina.imis.athena-innovation.gr/diana_tools/web/index.php?r=lncbasev2/index-predicted) or starBase v2.0 (http://starbase.sysu.edu.cn/), respectively. The dual luciferase assay was used to confirm the relationship of target genes. Specific steps were as follows: Firstly, the 3′UTR wild-type or mutant sequences of SLCO4A1-AS1 (SLCO4A1-AS1-wt or SLCO4A1-AS1-mut) or STAT3 (STAT3-wt or STAT3-mut) were recombined into pmirGLO vectors (CL414-01, Biomed, China). Next, the recombinant vectors were cotransfected with miR-149-5p mimic or blank into AGS or SNU-16 cells according to Lipofectamine 3000. Finally, the luciferase activity was measured using the dual luciferase reporter assay analysis system (D0010, Solarbio, China) and Promega GLOMAX 20/20 (USA), and the Renilla fluorescence value was used as the internal reference.

### 2.10. Western Blot

As mentioned in the literature previously [22], the total protein of AGS or SNU-16 cells was lysed and extracted by RIPA buffer containing 1% PMSF (R0010, Solarbio, China), and then the protein concentration was detected in the BCA kit (PC0020, Solarbio). After electrophoretic separation using sodium dodecyl sulfate-polyacrylamide gel electrophoresis (SDS-PAGE), the protein was transferred to a PVDF membrane (ISEQ00010) produced by Millipore (USA). Antibodies of STAT3 (ab119352, 88 kDa, 1/1000, Abcam, UK) or GAPDH (ab8245, 36 kDa, 1/1000) were added to the membranes and incubated at 4°C for 16 h. Afterwards, the membranes were incubated with the Goat Anti-Mouse (ab205719, 1/5000) for 2 h, followed by exposure with ultrasensitive ECL chemiluminescent substrate (SW2040, Solarbio, China). Lastly, LabWorks Image Acquisition and Analysis Software (UVP, Upland, USA) was used to analyze the data.

### 2.11. Statistical Analysis

By GraphPad Prism 8.0, independent-sample *t*-test or one-way ANOVA was performed to analyze the difference between two or among more groups. The correlation between target genes (SLCO4A1-AS1, miR-149-5p, STAT3) was evaluated by *Pearson* correlation coefficient. *P* < 0.05 was considered as statistically significant.

## 3. Results

The expression of SLCO4A1-AS1 was increased in gastric cancer and played a role as an oncogene.

**Figure 1:**
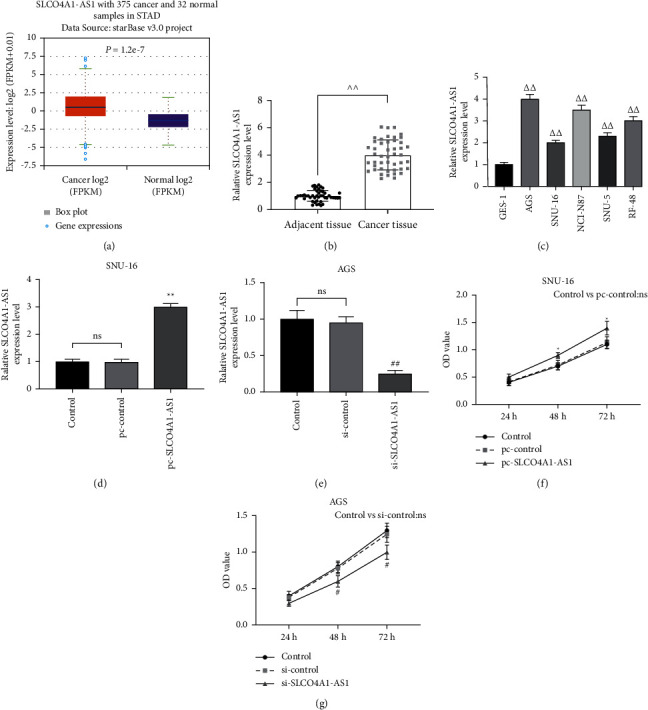
Highly expressed SLCO4A1-AS1 was detected in gastric cancer and regulated cell viability. (a) The starBase database (http://starbase.sysu.edu.cn/) analyzed the differential expression of SLCO4A1-AS1 in stomach adenocarcinoma (STAD) patients (*n* = 375) and normal samples (*n* = 32, *P*=1.2*e* − 7). (b, c) SLCO4A1-AS1 was highly expressed in gastric cancer tissues (*n* = 46) and cell lines, detected by RT-qPCR. (d, e) RT-qPCR was used to detect the level of SLCO4A1-AS1 after exogenous overexpression or intervention. (f, g) Overexpression of SLCO4A1-AS1 promoted SNU-16 cell viability, while silencing SLCO4A1-AS1 inhibited AGS cell viability, as determined by CCK-8. GAPDH was set as control. Each experiment was repeated three times independently. SLCO4A1-AS1: SLCO4A1 antisense RNA 1; RT-qPCR: Real-Time Quantitative PCR; CCK-8: Cell Counting Kit-8; ^^^^*P* < 0.01 versus adjacent tissue; ^△△^*P* < 0.01 versus GES-1; ^∗^*P* < 0.05 and ^∗^^∗^*P* < 0.01 versus pc-control; ^#^*P* < 0.05 and ^##^*P* < 0.01 versus si-control.

**Figure 2:**
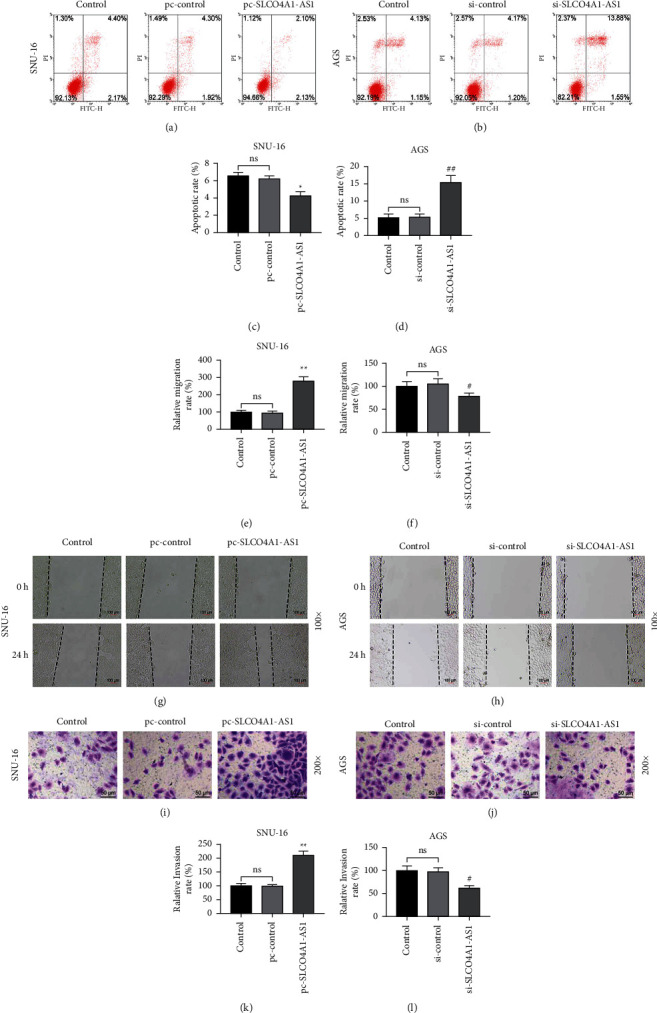
Silencing SLCO4A1-AS1, contrary to the overexpression of SLCO4A1-AS1, induced apoptosis and inhibited cell migration and invasion in gastric cancer. (a–d) Flow cytometry was used to detect the effect of SLCO4A1-AS1 exogenous overexpression or intervention on gastric cancer cell apoptosis. (e–h) The overexpression of SLCO4A1-AS1 promoted the migration of gastric cancer cells, while the low expression of SLCO4A1-AS1 had the opposite effect, as analyzed by the wound healing experiment (magnification ×100). (i–l) Transwell was used to detect the effect of SLCO4A1-AS1 overexpression or silencing on cell invasion ability (magnification ×200). Each experiment was repeated three times independently. ^*∗∗*^*P* < 0.05 and ^*∗∗*^*P* < 0.01 versus pc-control; ^*#*^*P* < 0.05 and ^*##*^*P* < 0.01 versus si-control.

The analysis of the starBase database showed that the expression of SLCO4A1-AS1 in stomach adenocarcinoma (STAD) patients (*n* = 375) was higher than that of normal samples (*n* = 32) (*P*=1.2*e* − 7, Figure 1(a)). We tested 46 gastric cancer tissues and matched paracancerous tissues and found that SLCO4A1-AS1 expression was upregulated in gastric cancer tissues (*P* < 0.01, Figure 1(b)). As shown in Figure 1(b), the expression of SLCO4A1-AS1 in AGS, SNU-16, NCI-N87, SNU-5, and RF-48 cell lines was significantly higher than that in GES-1 cell line (*P* < 0.01). Among them, the highest expression of SLCO4A1-AS1 was observed in AGS cells and the smallest expression of that is in SNU-16 cells (*P* < 0.01, Figure 1(c)). In order to investigate whether SLCO4A1-AS1 has an effect on the malignant phenotype of gastric cancer cells, SLCO4A1-AS1 was successfully induced to increase or suppress in gastric cancer cells (*P* < 0.01, Figures 1(d) and 1(e)). Then we found that overexpression of SLCO4A1-AS1 enhanced cell viability, inhibited apoptosis, and significantly accelerated cell migration and invasion in gastric cancer cells, while knockdown of SLCO4A1-AS1 had the opposite effect (*P* < 0.05, Figures 1(f), 1(g), and 2(a)–2(l)).

SLCO4A1-AS1 targeted and negatively regulated miR-149-5p, which was downregulated in gastric cancer tissues.

**Figure 3:**
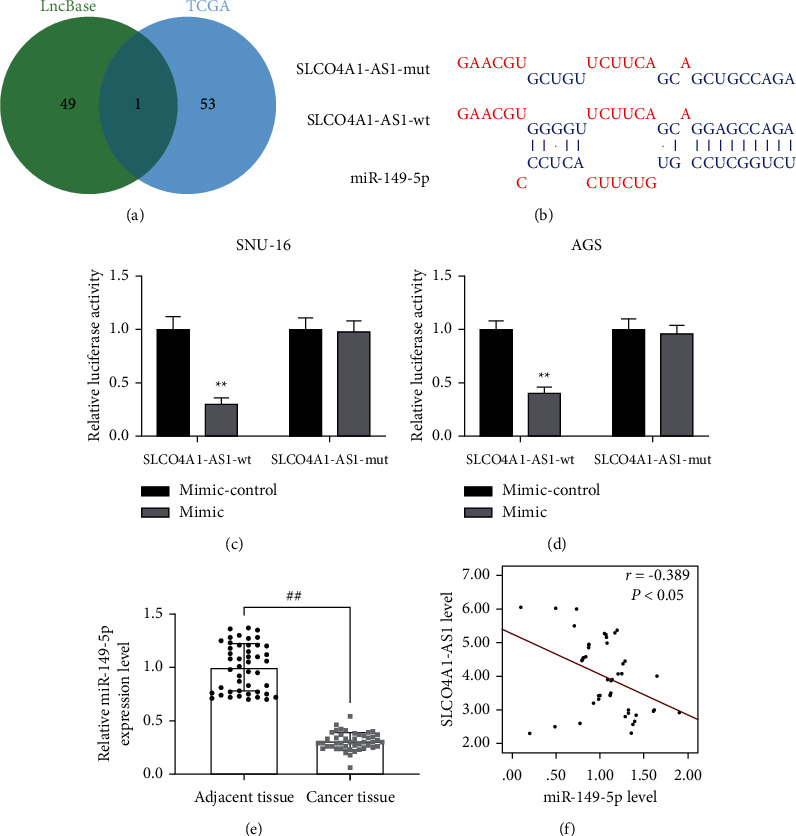
SLCO4A1-AS1 targeted and negatively regulated miR-149-5p, which was underexpressed in gastric cancer tissues. (a) The Venn diagram was used to obtain the intersection of the prediction results of the LncBase Predicted v.2 website (the targeted miRNA of SLCO4A1-AS1, http://carolina.imis.athena-innovation.gr/diana_tools/web/index.php?r=lncbasev2/index-predicted) and the specific low-expressed miRNA in STAD (provided by the TCGA database). (b–d) SLCO4A1-AS1 bound to miR-149-5p, predicted by LncBase Predicted v.2 website and verified by dual luciferase reporter gene. (e) RT-qPCR was used to detect the expression of miR-149-5p in gastric cancer tissues. (f) The *Pearson* correlation coefficient was used to analyze the relationship between miR-149-5p and SLCO4A1-AS1 in gastric cancer tissues (*r* = −0.389 and *P* < 0.05). Experiment was repeated three times independently. ^*∗∗*^*P* < 0.01 versus blank; ^*##*^*P* < 0.01 versus adjacent tissue.

In order to explore the mechanism of SLCO4A1-AS1 function in gastric cancer, we predicted the target miRNA of SLCO4A1-AS1. We predicted the miRNAs that SLCO4A1-AS1 can bind through the LncBase website, and after intersecting with the low-expressed miRNAs in the TCGA database, we obtained a common miRNA: miR-149-5p (Figure 3(a)). As shown in Figure 3(b), there was a binding site between miR-149-5p and SLCO4A1-AS1. Dual luciferase reporter gene results showed that, in SNU-16 and AGS cells, miR-149-5p could bind to the 3′UTR region of SLCO4A1-AS1 (*P* < 0.01, Figures 3(c) and 3(d)). In addition, the expression of miR-149-5p was suppressed in gastric cancer samples (*P* < 0.01, Figure 3(e)). Based on this, we tested the correlation between miR-149-5p and SLCO4A1-AS1 and found a negative correlation between the two (*r* = −0.389, *P* < 0.05, Figure 3(f)).

SLCO4A1-AS1 affected the biological function of gastric cancer cells through miR-149-5p.

**Figure 4:**
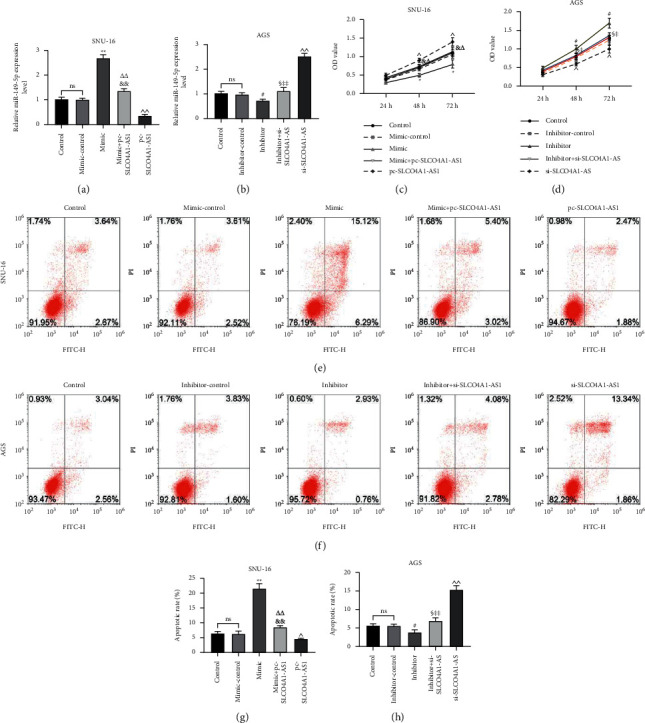
SLCO4A1-AS1 affected the viability and apoptosis of gastric cancer cells through miR-149-5p. (a) The expression of miR-149-5p in SNU-16 cells of control, mimic-control, mimic, mimic + pc-SLCO4A1-AS1, and pc-SLCO4A1-AS1 groups was detected by RT-qPCR. (b) The expression of miR-149-5p in AGS cells of control, inhibitor-control, inhibitor, inhibitor + si-SLCO4A1-AS1, and si-SLCO4A1-AS1 groups was detected by RT-qPCR. (c, d) miR-149-5p mimic inhibited cell viability, while inhibitor was the opposite, and miR-149-5p partially reversed the effect of SLCO4A1-AS1, as determined by CCK-8. (e–h) Upregulation of miR-149-5p promoted apoptosis, but downregulation was the opposite, and miR-149-5p partially reversed the role of SLCO4A1-AS1, as detected by flow cytometry. Each experiment was repeated three times independently. ^∗^*P* < 0.05 and ^∗∗^*P* < 0.01 versus mimic-control; ^&^*P* < 0.05 and ^&&^*P* < 0.01 versus mimic; ^△^*P* < 0.05 and ^△△^*P* < 0.01 versus pc-SLCO4A1-AS1; ^^^*P* < 0.05 and ^^^^*P* < 0.01 versus control; ^#^*P* < 0.05 versus inhibitor-control; ^§^*P* < 0.05 versus inhibitor; ^‡&^*P* < 0.05 and ^‡‡^*P* < 0.01 versus si-SLCO4A1-AS1.

**Figure 5:**
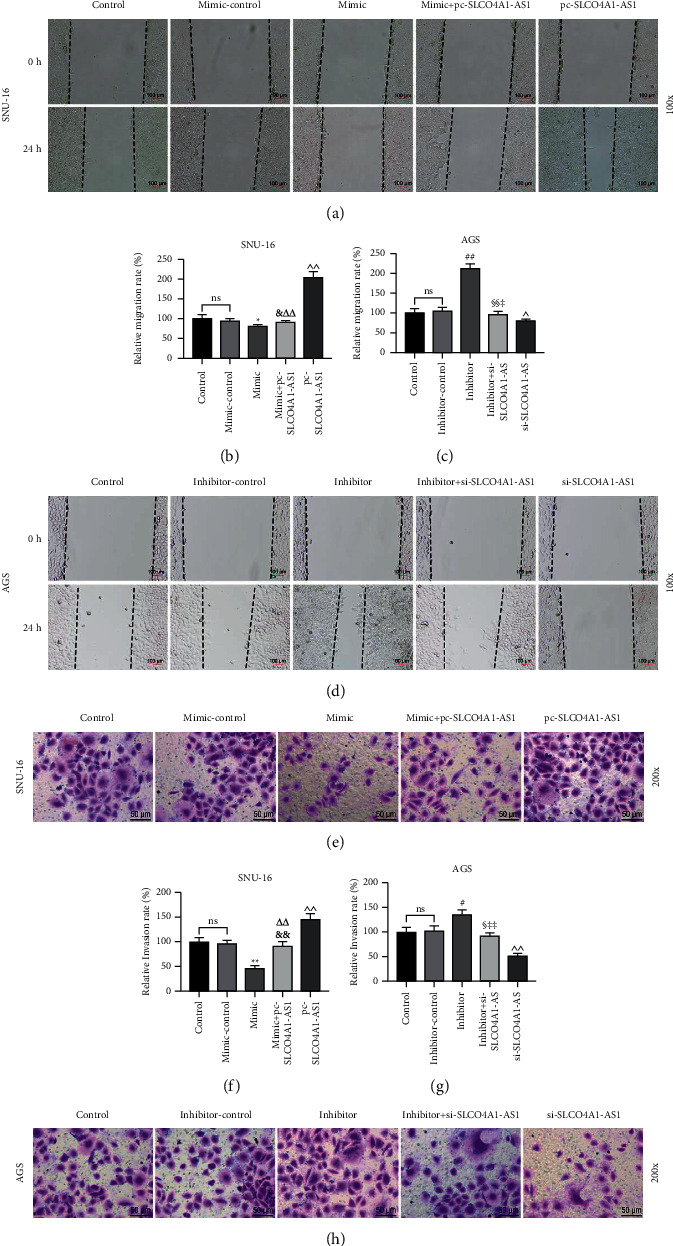
Effects of SLCO4A1-AS1/miR-149-5p axis on migration and invasion of gastric cancer cells. (a–d) miR-149-5p mimic inhibited cell migration, inhibitor was the opposite, and miR-149-5p partially reversed the effect of SLCO4A1-AS1. The results were analyzed by wound healing experiments (magnification ×100). (e, f) Invasion of SNU-16 cells in control, mimic-control, mimic, mimic + pc-SLCO4A1-AS1, and pc-SLCO4A1-AS1 groups was determined by Transwell (magnification ×200). (g, h) Invasion of AGS cells in control, inhibitor-control, inhibitor, inhibitor + si-SLCO4A1-AS1, and si-SLCO4A1-AS1 groups was measured by Transwell (magnification ×200). Each experiment was repeated three times independently. ^∗^*P* < 0.05 and ^∗∗^*P* < 0.01 versus mimic-control; ^&^*P* < 0.05 and ^&&^*P* < 0.01 versus mimic; ^△△^*P* < 0.01 versus pc-SLCO4A1-AS1; ^^^*P* < 0.05 and ^^^^*P* < 0.01 versus control; ^#^*P* < 0.05 and ^##^*P* < 0.01 versus inhibitor-control; ^§^*P* < 0.05 and ^§§^*P* < 0.01 versus inhibitor; ^‡^*P* < 0.05 and ^‡‡^*P* < 0.01 versus si-SLCO4A1-AS1.

Rescue experiments were conducted to investigate whether SLCO4A1-AS1 affects gastric cancer cell function through miR-149-5p. As shown in Figures 4(a) and 4(b), overexpression of SLCO4A1-AS1 significantly inhibited miR-149-5p levels induced by miR-149-5p mimic, while knockdown of SLCO4A1-AS1 increased miR-149-5p levels suppressed by miR-149-5p inhibitor (*P* < 0.05). miR-149-5p mimic reduced cell viability, induced apoptosis, and significantly reduced cell migration and invasion, while miR-149-5p inhibitor had the opposite effect in gastric cancer cells (*P* < 0.05, Figures 4(c)–4(h) and 5(a)–5(h)). Moreover, the mimic + pc-SLCO4A1-AS1 group significantly reversed the effect of the mimic group or pc-SLCO4A1-AS1 group, and the inhibitor + si-SLCO4A1-AS1 group significantly neutralized the effect of the inhibitor group or si-SLCO4A1-AS1 group (*P* < 0.05, Figures 4(c)–4(h) and 5(a)–5(h)).

STAT3 may be the downstream target gene of SLCO4A1-AS1/miR-149-5p axis.

**Figure 6:**
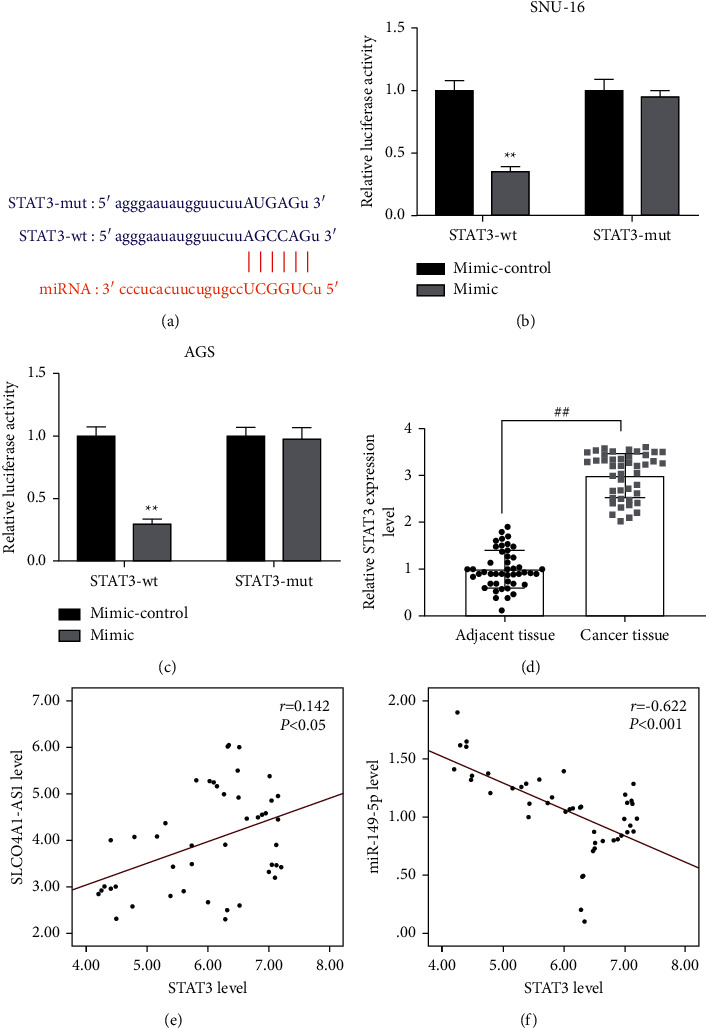
The regulatory relationship and expression of SLCO4A1-AS1-miR-149-5p-STAT3 in gastric cancer. (a–c) STAT3 was the target gene of miR-149-5p, which was predicted by starBase database (https://starbase.sysu.edu.cn/) and verified by dual luciferase reporter gene. (d) STAT3 was elevated in gastric cancer tissues, detected by RT-qPCR (*n* = 46). Experiment was repeated three times independently. (e, f) *Pearson* correlation coefficient was used to analyze the relationship between STAT3 and SLCO4A1-AS1 (*r* = 0.142 and *P* < 0.05), as well as STAT3 and miR-149-5p (*r* = −0.622 and *P* < 0.001). Each experiment was repeated three times independently. ^*∗∗*^*P* < 0.01 versus blank; ^*##*^*P* < 0.01 versus adjacent tissue. STAT3, signal transducer and activator of transcription 3.

As shown in Figure 6(a), STAT3 was targeted by miR-149-5p. The luciferase activity of miR-149-5p mimic was obviously inhibited in STAT3-wt group, while there was no significant difference of that in STAT3-mut group (*P* < 0.01, Figures 6(b) and 6(c)). We found that the expression of STAT3 in cancer tissues was significantly abnormally upregulated (*P* < 0.01, Figure 6(d)). Then we tested the correlation between STAT3 and SLCO4A1-AS1 or miR-149-5p. The results showed that the expressions of STAT3 and SLCO4A1-AS1 were positively correlated in gastric cancer tissues (*r* = 0.142, *P* < 0.05, Figure 6(e)), but STAT3 was negatively correlated with miR-149-5p (*r* = -0.622, *P* < 0.001, Figure 6(f)). In addition, we further examined the protein content of STAT3 and found that STAT3 expression was decreased in the mimic group but was increased in the pc-SLCO4A1-AS1 group, and the mimic + pc-SLCO4A1-AS1 group significantly counteracted the effect of mimic group or pc-SLCO4A1-AS1 group on STAT3 expression (*P* < 0.05, Supplementary Figures 1(a)–1(c) and Supplementary Figure 2(a)). Conversely, STAT3 expression was increased in the inhibitor group but was decreased in the si-SLCO4A1-AS1 group, and the inhibitor + si-SLCO4A1-AS1 group significantly reversed the inhibitory effect of the inhibitor group or si-SLCO4A1-AS1 group on STAT3 expression (*P* < 0.01, Supplementary Figures 1(d)–1(f) and Supplementary Figure 2(b)).

## 4. Discussion

There are a large number of regulatory interaction sites in lncRNAs, which provides a broader platform for the development of new structure-based anticancer drugs [23]. In addition, in view of their involvement in multiple cell signaling pathways and tissue-specific expression, lncRNAs can also be used in new strategies for the diagnosis and targeting of specific cancer subtypes [24]. This study found that SLCO4A1-AS1 has a cancer-promoting effect in gastric cancer cells, which may regulate the biological characteristics of gastric cancer cells by targeting STAT3 with miR-149-5p, suggesting that SLCO4A1-AS1 may be used for the treatment of gastric cancer.

Gastric cancer, as a malignant tumor of the digestive tract with a high mortality rate, has already attracted the attention of scholars at home and abroad [25]. It is necessary to study the molecular mechanism of gastric cancer, hoping to provide effective assistance for the treatment of gastric cancer. For example, LncRNA-PCTA6 participates in the endogenous competition of miR-30 by targeting the Makorin RING finger protein 3 to promote the malignant transformation of gastric cancer [26]. Wang et al. revealed that the expression of lncRNA AB007962 is downregulated in gastric cancer tissues, and the expression level is negatively correlated with tumor size; furthermore, it indicates that the expression of AB007962 is significantly correlated with poor prognosis [27]. Our research revealed that SLCO4A1-AS1 is elevated in gastric cancer, which is similar to the expression trend of LncRNA-PCTA6 [26], so we speculate that SLCO4A1-AS1 may also have a role in promoting malignant transformation of gastric cancer. SLCO4A1-AS1 has different roles in different cancers, but the cell experiments in this study proved our conjecture, showing that pc-SLCO4A1-AS1 promotes cell viability, migration, and invasion and inhibits apoptosis, while si-SLCO4A1-AS1 inhibits cell growth and migration and promotes apoptosis. It suggests that SLCO4A1-AS1 may be involved in the growth and metastasis of gastric cancer tumors, and our results are consistent with the research of Ouyang et al. [28]. Compared with the sudden rise of lncRNA research, miRNA has always been the focus of cancer research [29]. Therefore, the target miRNA based on bioinformatics is essential to more fully reveal the tumor-related pathways involved in lncRNA. We experimentally verified that SLCO4A1-AS1 can bind to miR-149-5p.

There have also been numerous reports of miRNA research in gastric cancer. Recently, the expression of miR-582 has been found to increase in gastric cancer, and it may activate PI3K/AKT/Snail axis through FOXO3 to regulate the proliferation and metastasis of gastric cancer cells [30]. miR-149-5p is one of the two active chains produced by miR-149 precursors, which can act on their respective target genes to exert biological effects. The role of miR-149-5p in a variety of diseases has been reported, including various cancers [31, 32], atherosclerosis [33], and diabetes [34]. The regulation is mostly related to the sponge mechanism of lncRNA-miRNA. Consistent with the report of Zhang et al. [35], our results confirmed the downregulation of miR-149-5p in gastric cancer and the inhibition of cancer cell function. The difference is that the research team first revealed that SLCO4A1-AS1 can regulate the malignant phenotype of gastric cancer cells through miR-149-5p.

To the best of our knowledge, miRNAs can regulate the upregulation or downregulation of mRNA by targeting mRNA and then activate related pathways to play a role in cancer cells [36]. Mechanically, we explained that STAT3 is a downstream target gene of miR-149-5p and that the two have a negative regulatory relationship. Many studies have shown that STAT3 plays an important role in JAK/STAT, promoting cell proliferation, migration, and invasion [37]. Wu et al. explored that cancer-associated fibroblasts can facilitate gastric cancer migration and EMT in the tumor microenvironment by IL-6/JAK2/STAT3 signaling [38]. Although we demonstrated that SLCO4A1-AS1 positively regulates STAT3 through sponging miR-149-5p in gastric cancer cells, the downstream regulatory mechanism of STAT3 in gastric cancer still needs to be verified. Our next work may focus on *in vivo* functional verification and more regulatory networks.

## 5. Conclusion

To sum up, our research reveals that SLCO4A1-AS1 may explain part of the progression of gastric cancer and may target STAT3 through competitive binding of miR-149-5p to affect the growth and metastasis of gastric cancer tumors.

## Data Availability

The analyzed data sets generated during the study are available from the corresponding author upon reasonable request.

## Supplementary Materials

Supplementary Materials S1: Western blot and RT-qPCR were used to detect the expression of STAT3 in SNU-16 cells and AGS cells. Each experiment was repeated three times independently. *∗∗P* < 0.01 versus mimic-control; ^&&^*P* < 0.01 versus mimic; ^△△^*P* < 0.01 versus pc-SLCO4A1-AS1; ^^^*P* < 0.05 and ^^^^*P* < 0.01 versus control; ^##^*P* < 0.01 versus inhibitor-control; ^§§^*P* < 0.01 versus inhibitor; ^‡‡^*P* < 0.01 versus si-SLCO4A1-AS1. S2: the original experimental results of Western blot in S1.
