# Supplementary figures and images for: SLCO4A1-AS1 Facilitates the Malignant Phenotype via miR-149-5p/STAT3 Axis in Gastric Cancer Cells
Source: J Oncol. 2021 Oct 19;2021:1698771. doi: 10.1155/2021/1698771 (PMC8548156; doi:10.1155/2021/1698771)

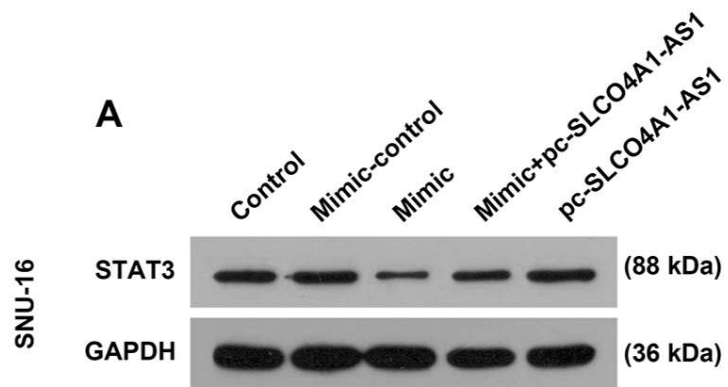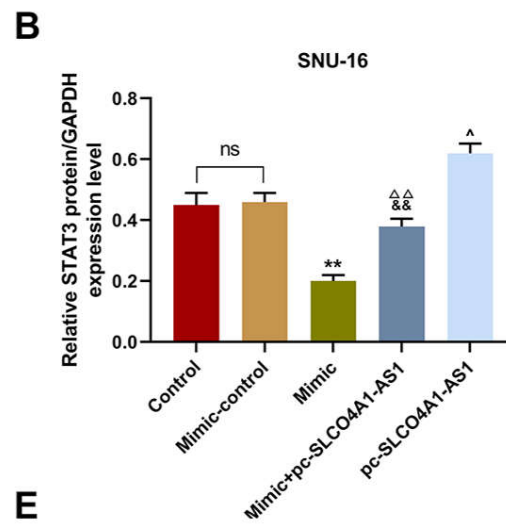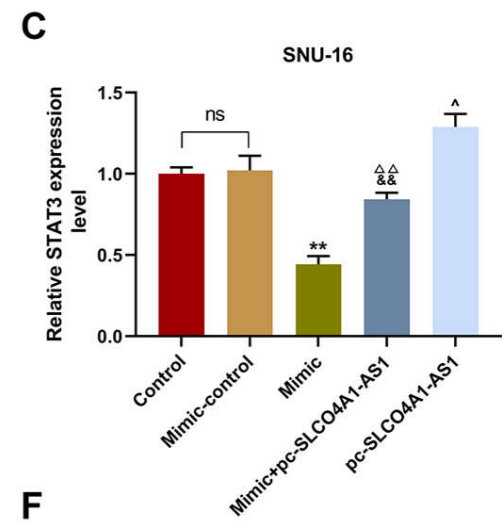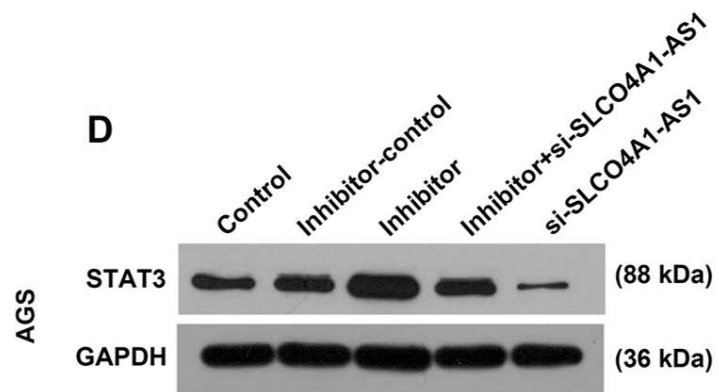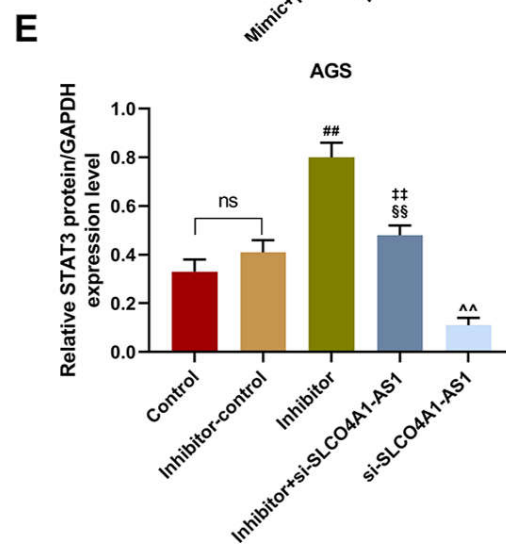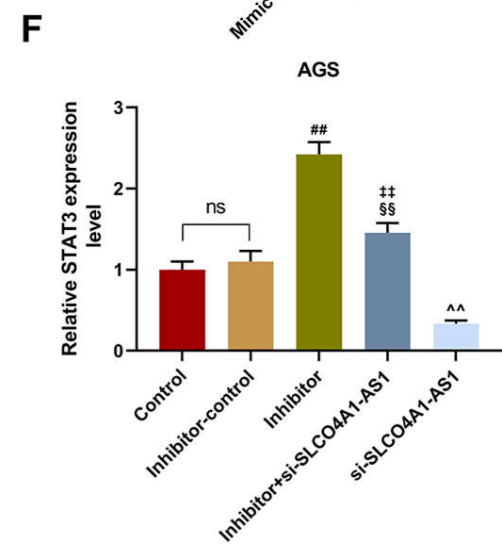

Supplement: Supplementary Materials — S1: Western blot and RT-qPCR were used to detect the expression of STAT3 in SNU-16 cells and AGS cells. Each experiment was repeated three times independently. ∗∗P < 0.01 versus mimic-control; &&P < 0.01 versus mimic; △△P < 0.01 versus pc-SLCO4A1-AS1; ^P < 0.05 and ^^P < 0.01 versus control; ##P < 0.01 versus inhibitor-control; §§P < 0.01 versus inhibitor; ‡‡P < 0.01 versus si-SLCO4A1-AS1. S2: the original experimental results of Western blot in S1. [file 1698771.f1.zip › 1698771.f1/Supplementary figure 1.pdf]

A

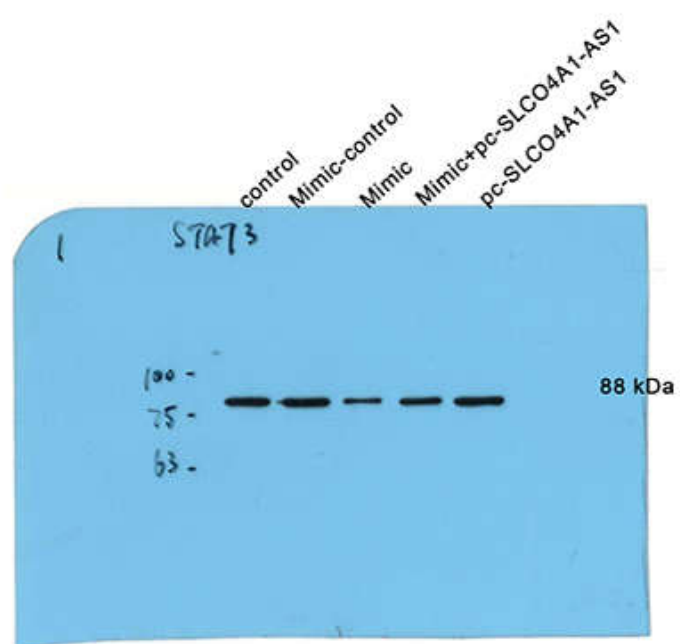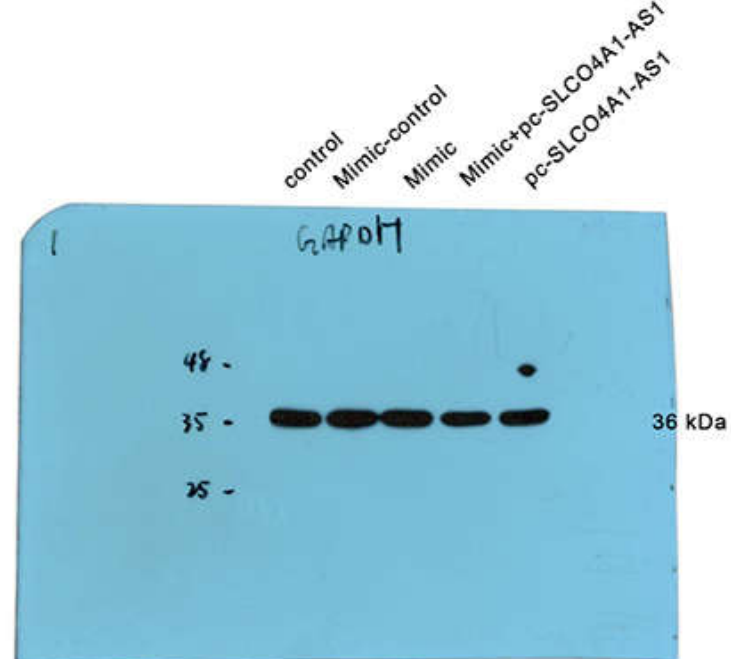

B

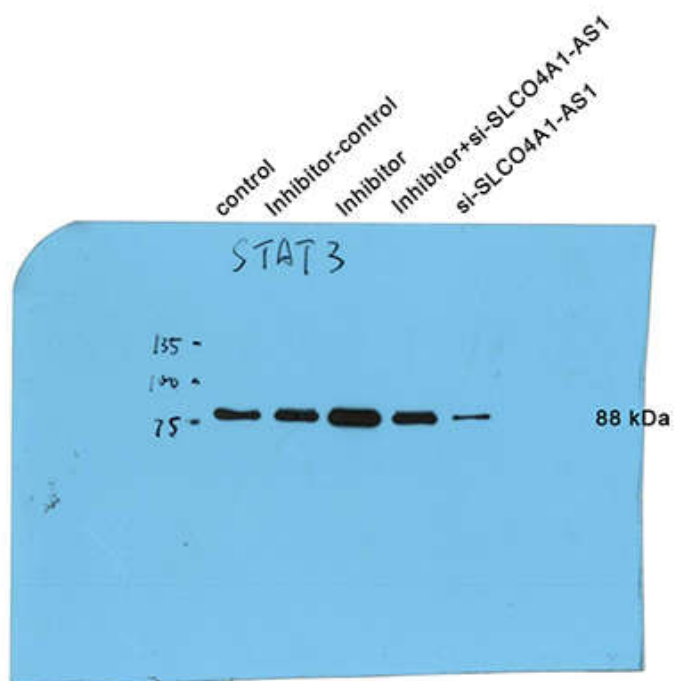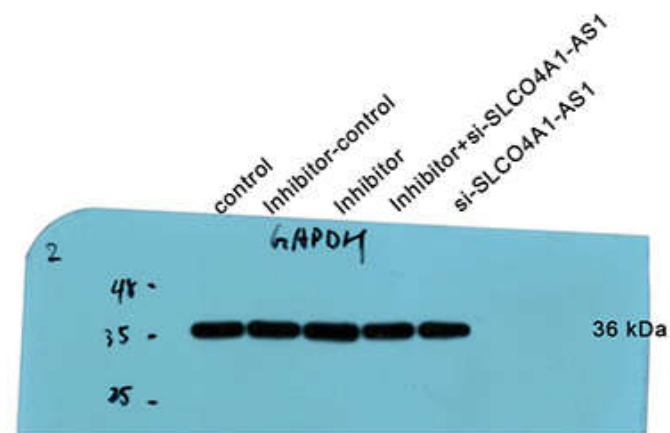

Supplement: Supplementary Materials — S1: Western blot and RT-qPCR were used to detect the expression of STAT3 in SNU-16 cells and AGS cells. Each experiment was repeated three times independently. ∗∗P < 0.01 versus mimic-control; &&P < 0.01 versus mimic; △△P < 0.01 versus pc-SLCO4A1-AS1; ^P < 0.05 and ^^P < 0.01 versus control; ##P < 0.01 versus inhibitor-control; §§P < 0.01 versus inhibitor; ‡‡P < 0.01 versus si-SLCO4A1-AS1. S2: the original experimental results of Western blot in S1. [file 1698771.f1.zip › 1698771.f1/Supplementary figure 2.pdf]
